# Basolateral amygdala to posterior piriform cortex connectivity ensures precision in learned odor threat

**DOI:** 10.1038/s41598-021-01320-4

**Published:** 2021-11-05

**Authors:** Brett S. East, Gloria Fleming, Samantha Vervoordt, Prachi Shah, Regina M. Sullivan, Donald A. Wilson

**Affiliations:** 1 Emotional Brain Institute, Nathan Kline Institute for Psychiatric Research, Orangeburg, NY USA; 2 Child and Adolescent Psychiatry, New York University Langone Medical Center, 1 Park Avenue, 7th Floor, New York, NY 10016 USA

**Keywords:** Olfactory cortex, Olfactory system, Amygdala

## Abstract

Odor perception can both evoke emotional states and be shaped by emotional or hedonic states. The amygdala complex plays an important role in recognition of, and response to, hedonically valenced stimuli, and has strong, reciprocal connectivity with the primary olfactory (piriform) cortex. Here, we used differential odor-threat conditioning in rats to test the role of basolateral amygdala (BLA) input to the piriform cortex in acquisition and expression of learned olfactory threat responses. Using local field potential recordings, we demonstrated that functional connectivity (high gamma band coherence) between the BLA and posterior piriform cortex (pPCX) is enhanced after differential threat conditioning. Optogenetic suppression of activity within the BLA prevents learned threat acquisition, as do lesions of the pPCX prior to threat conditioning (without inducing anosmia), suggesting that both regions are critical for acquisition of learned odor threat responses. However, optogenetic BLA suppression during testing did not impair threat response to the CS+ , but did induce generalization to the CS−. A similar loss of stimulus control and threat generalization was induced by selective optogenetic suppression of BLA input to pPCX. These results suggest an important role for amygdala-sensory cortical connectivity in shaping responses to threatening stimuli.

## Introduction

Central odor processing and odor perception occur in the context of both the multisensory environment and internal state. Thus, multisensory visual and auditory cues can affect how odors are perceived, with for example, red liquids being perceived as more cherry-like than clear liquids^1–3^. This multisensory modulation of perception is associated with changes in underlying central processing of odors, especially in piriform cortex (PCX)^4–8^. Furthermore, internal states such as hunger, sleep/wake cycle, and task demands can affect odor perception and coding^9–13^, again in many instances with the PCX as a critical site for such modulation^5,13–18^.

Similarly, while odors can evoke hedonic or emotional states^12,14,19,20^, emotions, as internal states, can also modulate odor perception and coding^21^ suggesting a bidirectional relationship between these basic processes^9,22^. For example, learned association between odors and hedonically valenced events such as rewards or threats can modify neural activity and responses to those learned odors in the PCX as well as behavioral response to the odors^15,23–31^.

Although large brain networks contribute to threat and reward processing and emotion, the amygdala complex has been strongly linked to emotional affect and learning, including threat assessment, action, fear, and reward, appearing to function as a multisensory integrator of high valence information^32–38^. Of relevance to olfaction, neurons in the basolateral amygdala (BLA) are necessary for odor threat conditioning^39^, respond to odors^40^ and odor-evoked responses in both the BLA^41,42^ and PCX^26^ are modified by odor-threat conditioning, as are odor-evoked oscillations recorded in local field potentials (LFP)^28,33,43^. The amygdala complex receives direct input from the olfactory bulb^44^, and there are strong reciprocal connections between the PCX and several amygdala nuclei, with especially strong connections between the BLA and the posterior piriform cortex (pPCX)^45^. Optogenetic activation of BLA projections to the pPCX activates both pPCX interneurons and pyramidal cells in vitro^46^, providing a direct BLA impact on pPCX odor coding circuits. In fact, optogenetic activation of BLA projections to the pPCX in vivo modifies both single-unit and single-unit ensemble coding of odors^47^.

Context or learned changes in odor coding can have several consequences with adaptive significance for responding to threatening stimuli. These could include changes in response threshold or intensity which could enhance signal:noise and thus detectability of the threat^48,49^, and/or changes in acuity which could help direct responses to specific stimuli and prevent generalization^26,50^. Previous work has demonstrated that, depending on the threat conditioning protocol, animals can learn either odor-specific threat responses (e.g., discriminative threat/safety signal training) or generalized threat responses (e.g., single stimulus threat training), and that PCX single-units narrow or broaden their odor receptive fields in line with the learned behavioral response^26^. Here, we hypothesize that input from the BLA to the pPCX provides an instructive signal to the pPCX to allow these alternative behavioral outcomes. Using a variety of techniques, we demonstrate (1) that functional connectivity between the BLA and pPCX is enhanced after differential threat conditioning, (2) that both the BLA and pPCX are required for acquisition of odor threat conditioning, and (3) that selective suppression of the BLA projection to pPCX during expression of learned odor threat induces loss of stimulus control and, instead, expression of generalized odor-induced threat responses to both the threat and safety odors. These results suggest an important role for amygdala-sensory cortical connectivity in shaping responses to threatening olfactory stimuli.

## Methods

Male, Long-Evans rats (200–450 g) from Envigo Lab Animals were used. Animals were housed in polypropylene cages with ad libitum access to food and water. Cages were located within an animal facility that was both temperature and humidity controlled with a 12 h on:off cycle. All experiments were conducted during the lights-on period. There were a total of 107 rats (electrophysiology n = 26; lesions, n = 14, optogenetics n = 67). All animal procedures were performed with the Nathan S. Kline Institute for Psychiatric Research Institutional Animal Care and Use Committee’s approval and were in accord with National Institutes of Health guidelines. Animal use follows the recommendations of the ARRIVE guidelines^51^.

For differential odor threat conditioning, animals were placed in a Plexiglas chamber (24 × 24 × 36 cm; width × length × height) surrounding a grid floor (Lafayette instrument, Lafayette, IN). One side of the chamber included a port with odor delivery from a computer controlled olfactometer. A vacuum was placed on the opposite side of the chamber to draw odors across the chamber and subsequently remove them between presentations. A partial barrier was placed over the top of the chamber to prevent escape. The barrier had a central hole to allow video recording. Activity within the chamber was recorded with a video camera above the chamber and an angled mirror along one side of the chamber to allow top and side viewing. Animals were habituated to handling and the chamber for at least 2 days prior to training. During training, the animals received 10 presentations of the CS+ odor (vanilla extract; McCormick & Co., Inc, Hunt Valley, MD) and 30 presentations of the CS− odor (peppermint extract; McCormick & Co., Inc, Hunt Valley, MD). Trials were presented pseudo-randomly with a 120 s ITI. For all trials, the 15 s CS+ or CS− odor was presented under the control of a flow-dilution olfactometer at 1LPM controlled by Spike2. For CS+ trials, the final second of the odor overlapped with a one second footshock (0.5 mA) with both co-terminating. To control for the possibility of the sound of the olfactometer switching on and off being used as a predictive cue, activation of an empty valve containing no odor were also pseudorandomly dispersed (5 s duration, n = 23) throughout the length of each session.

Testing occurred 24 h following training in the same chamber as training, but with the visual context altered: the clear Plexiglas walls were covered with a black and white checkerboard pattern, the grid floor was covered by a hard plastic floor, and the roof of the chamber was left open. The CS+ and CS− odors (15 s duration) were presented 3 times in pseudorandom order with at least a 3 min ITI. In the lesion experiment, animals were also presented with a novel odor (10% isoamyl acetate [Sigma], 15 s) during testing. The amount of time spent freezing (no movement, crouching posture) was quantified for all trials for each odor during testing.

For recording of local field potentials (LFP), implants capable of recording LFP from two channels simultaneously (ETA-F20 transmitters, Data Sciences International) were implanted subcutaneously under isoflurane anesthesia with stainless steel electrodes (120 µm) targeting the ipsilateral BLA and pPCX (BLA: AP − 3.0 mm, ML ± 4.9 mm from bregma, DV − 7.0 mm from brain surface; pPCX: AP − 1.5 mm, ML ± 5.6 mm, DV − 9 mm from bregma). The electrodes and reference were secured to the skull with dental cement, and the scalp sutured. Rats received injections of the analgesic BuprenorphineSR (0.1 mg/kg, s.c. injection) and the antibiotic Enrofloxacin (5 mg/kg s.c.) prior to recovery. Animals were allowed to recover 1 week prior to handling and differential odor threat training. Transmitted recordings were acquired with a receiver under the training chamber and digitized (10 kHz) using Spike2 software (Cambridge Electronic Design, LTD). Recordings were made throughout training and testing, as well as during a 0.5–1 h home cage spontaneous activity session. Spontaneous and stimulus evoked activity were analyzed in both the BLA and pPCX with FFT (2.4 Hz bins, Hanning window in Spike2 software [CED, Inc]). In addition, coherence between BLA and pPCX activity was assessed with the cohere script in Spike2 with a resolution of 2.4 Hz. Odor-evoked activity was specifically assessed during the first 5 s of odor presentation. Full spectrum (0–100 Hz) and high gamma band (61–90 Hz) activity were used for analyses as described in the Results. Following all testing, animals were perfused and electrode positions within the BLA and pPCX confirmed.

Excitotoxic lesions of the pPCX were induced with ibotenic acid in some animals. Under isoflurane anesthesia, rats received bilateral infusions at three sites in the pPCX, each consisting of 2.5 µg of ibotenic acid dissolved in 0.25µL PBS or 0.25µL PBS alone as a control. The infusion cannula was lowered to the desired site and allowed to infuse at a rate of 0.25µL/min for 5 min. After the infusion was complete, the cannula was left in place an additional 10 min to allow for diffusion. Rats received infusions at 3 sites bilaterally for a total of 6 injection sites at the following coordinates (all relative to bregma in mm) as shown in Table 1.

**Table 1:** Coordinates for ibotenic acid infusions into the pPCX to all extended damage across the rostral-caudal extent.

Plane	Site 1	Site 2	Site 3
AP	− 0.8	− 2.0	− 3.2
ML	± 5.4	± 5.6	± 5.8
DV	− 9.2	− 9.8	− 10.0

Following surgery, rats received injections of the analgesic BuprenorphineSR (0.1 mg/kg, s.c. injection), the antibiotic Enrofloxacin (5 mg/kg s.c.), and an i.p. injection of 0.2 mL diazepam (5 mg/mL) prior to recovery. Following a week of recovery, rats began handling and differential odor threat training as described above. At least 2 days following odor threat testing, animals were tested for anosmia with the buried food test^52^. Rats were placed in a clean standard housing cage with a familiar bacon-scented food pellet buried under the wood chip bedding. The latency to find the food was scored, with a 300 s maximum session length. In addition, rats were tested in an open field arena (black arena 66 × 66 × 38 cm, W × L × H) to assess hyper/hypo-activity that may have been induced by the lesions. Total open field session duration was 300 s. Following all tests, animals were perfused and lesions reconstructed to confirm localization to the pPCX.

For optogenetic manipulations, rats received bilateral infusions of AAV5-CaMKIIa-eArchT3.0-eYFP (ArchT, 3 × 10^12^ IU/mL) under isoflurane anesthesia. Bilateral holes were drilled (AP − 3.0 mm, ML ± 4.9 mm from bregma) for cannula access. The infusion cannula was lowered to the desired site (DV − 8.0 mm from brain surface) and used to infuse at a rate of 0.1µL/min for 3 min. Once infusion was completed, the cannula was left in place an additional 10 min to allow for diffusion. For optogenetic control (e.g., viral expression, light-induced tissue heating, etc.) animals were infused with AAV5-CaMKIIa-eYFP (2.5 × 10^12^ IU/mL; UNC Vector Core) in the same manner.

During the same surgery, rats had optical fibers (200 µm diameter, Doric) implanted. Optical fibers were targeted bilaterally at either the BLA or the pPCX. For those targeting the BLA, implant coordinates were AP − 3.0 mm, ML ± 4.9 mm from bregma, DV − 7.0 mm from brain surface. For those targeting pPCX, implant coordinates were AP − 1.5 mm, ML ± 5.6 mm, DV − 9 mm all from bregma. The fiber implant was secured to the skull with dental cement and bone screws. The scalp was sutured around the exposed optical fiber headcap. Rats received injections of the analgesic BuprenorphineSR (0.1 mg/kg, s.c. injection) and the antibiotic Enrofloxacin (5 mg/kg s.c.) prior to recovery and given at least 7 days for recovery.

Animals were trained and tested in the differential odor threat paradigm described above. Prior to training, animals were habituated to having their optical fiber implants connected to optical cables which were connected to a 532 nm laser (Shanghai Laser) through an optical swivel (Doric) which allowed free movement within the chamber. For optical suppression of the BLA or BLA projections selectively to the pPCX the laser was turned on (> 8mW) continuously beginning 1 s prior to onset of the CS+ odor and ramped down over 200 ms starting at the end of the odor/shock, similarly to previously described^53^. This ramp down reduces excitatory rebound that can occur following prolonged suppression^54^. The random 5 s noise stimuli that occurred during training to reduce significance of potential olfactometer noise was also associated with laser stimulation to reduce the behavioral significance of light flashes. Additional optogenetic controls included viral control as described above and animals that were connected to optical cables but had no light stimulation (see “Results”). For those rats receiving optogenetic suppression during testing, the same level and timing of light evoked suppression was instead applied during both CS+ and CS− odor presentations during the testing phase. Following testing, animals were perfused and viral infections limited to the bilateral BLA were confirmed.

Following the completion of testing, rats were overdosed with urethane (3 g/kg) prior to transcardial perfusion with PBS followed by 4% paraformaldehyde. Brains were extracted and stored in 20% glycerol prior to being sliced at 40 µm coronal sections for histological verification of lesion, electrode, fiber optic and viral infection locations. Virus expression was amplified with immunohistochemistry for YFP and confirmed using standard fluorescent microscopy or confocal microscopy.

Data were analyzed with two-way ANOVA across conditions, trials and stimuli as applicable, and appropriate post-hoc comparisons, using Prism 9 software. Additional details of statistical comparisons are provided with each assay in the Results.

## Results

**Figure 1:**
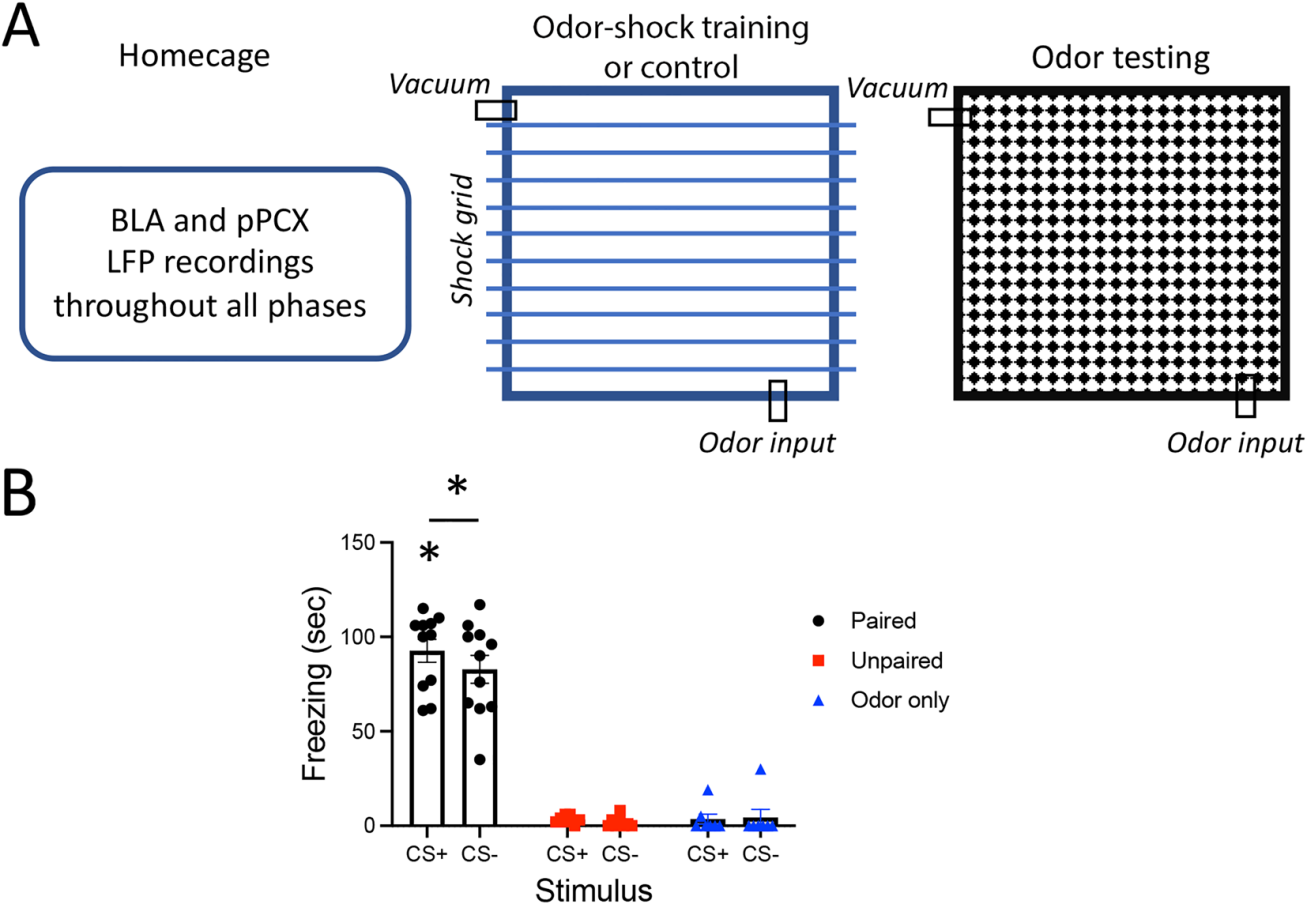
(**A**) Rats were implanted with electrodes in the BLA and pPCX for LFP recordings of odor-evoked and spontaneous local oscillations and BLA-pPCX coherence in the homecage, during odor-shock or control conditioning, and odor-evoked responses 24 h later in a different context. (**B**) Odor threat conditioning evoked freezing to the CS+ odor and significantly less freezing to the CS− odor (carat signifies CS+ evoked freezing was significantly more than CS− evoked freezing, *p* < 0.05). Paired animals froze significantly more than un-shocked Controls (asterisks signify *p* < 0.05).

Functional connectivity between pPCX and BLA was enhanced during acquisition and expression of learned odor threat. Rats were implanted with electrodes targeting the BLA and ipsilateral pPCX for telemetered simultaneous LFP recordings. Animals (Paired, n = 11) were trained in a differential odor threat conditioning paradigm, with one odor (CS+ , odor = vanilla extract) predicting footshock and another (CS−, odor = peppermint extract) predicting no footshock (i.e., safety)^33^. Two control groups included an Unpaired group that received the same number of footshocks and odor stimuli but with no association between the odors and shock (n = 8) and an Odor only control (n = 7) that was exposed to the same odor stimulation protocol, but no footshocks were delivered. Twenty-four hrs later, odor-evoked freezing was assessed in a novel context. Spontaneous and odor-evoked LFP recordings were obtained throughout the training and testing procedure, as well as spontaneous LFPs in the homecage (Fig. 1A).

Differential threat conditioning induced odor-specific freezing (Fig. 1B) in the Paired animals compared to both controls (3 × 2, group X odor ANOVA, main effect of group, F(2,23) = 113.2, *p* < 0.0001). A post-hoc t-test revealed the Paired animals froze significantly more to the CS+ than to the CS− (t(10) = 2.05, *p* = 0.033).

**Figure 2:**
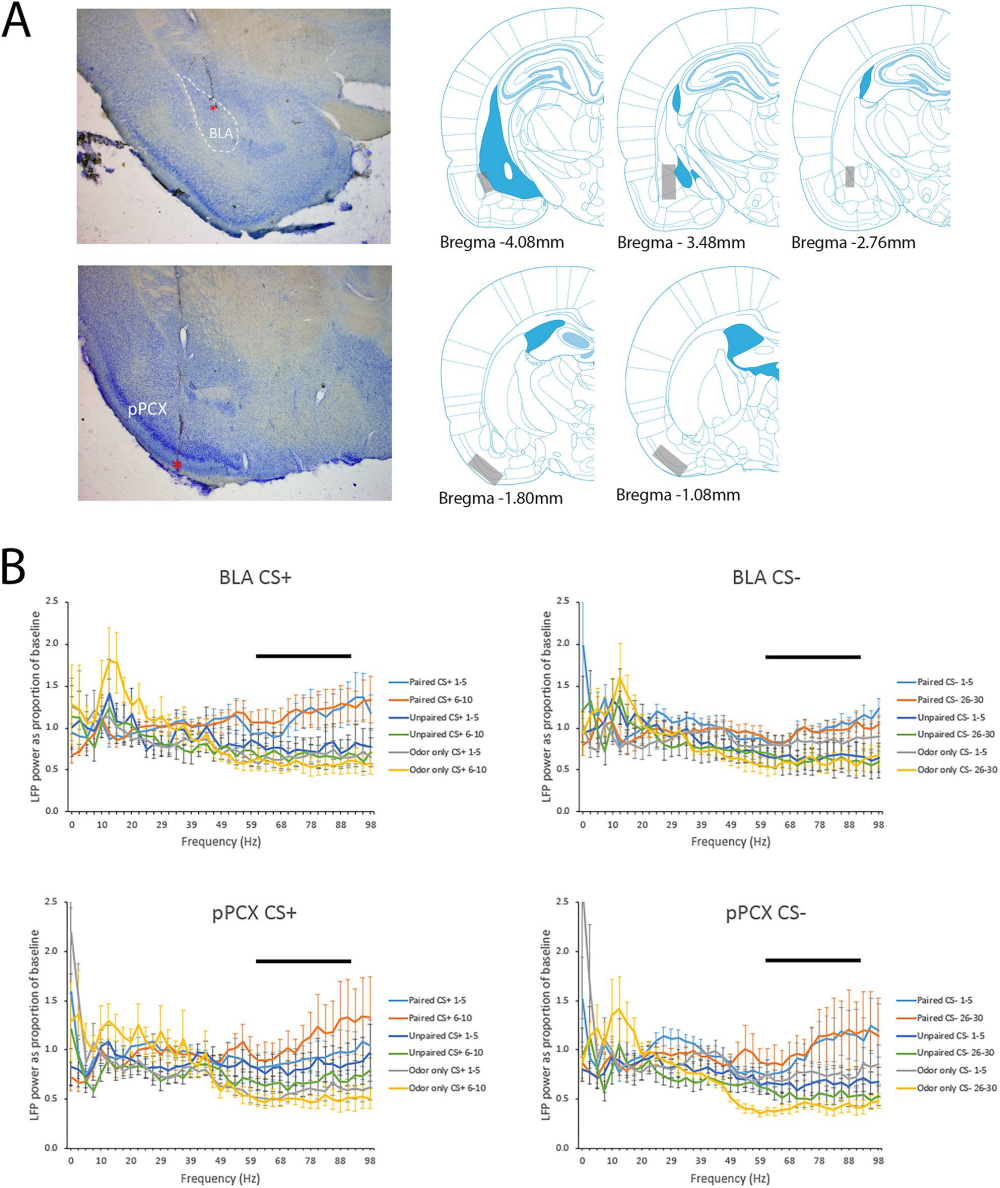
(**A**) Examples of electrode implants (red asterisks) in the BLA and pPCX and regional localization across animals of targeted areas. Schematic brain images were modified from Paxinos and Watson^77^. (**B**) Mean FFT’s normalized to spontaneous activity recordings taken in the homecage from the BLA and pPCX in response to the CS+ and CS− odors in Paired, Unpaired and Odor only animals. Mean activity is plotted over the first 5 and last 5 training trials for each odor in each location. The most robust differences between Paired and control groups were in the high gamma band (61–90 Hz), highlighted by the horizontal bar.

Responses to the CS+ and CS− odors were examined during the first 5 s of stimulation in both the BLA and pPCX with FFT analyses expressed relative to baseline oscillatory activity recorded in the home cage. Two animals in the Paired group had poor quality recordings (large movement artifacts) and were not included in the data analysis, leaving n = 9. Two animals in the Unpaired group similarly had large movement artifacts in both channels and were removed, leaving n = 6. For statistical data analyses, the mean power was calculated across the first 5 trials of both the CS+ and CS− (trials 1–5), and the last 5 trials of the CS+ (trials 6–10) and CS− (trials 26–30). Odor evoked activity was also examined during testing as the mean of the 3 CS+ and CS− novel context trials. As shown in Fig. 2, with this paradigm the most robust training-associated changes in oscillatory activity relative to control animals were in high gamma frequency band (61–90 Hz) in both the BLA and pPCX.

**Figure 3:**
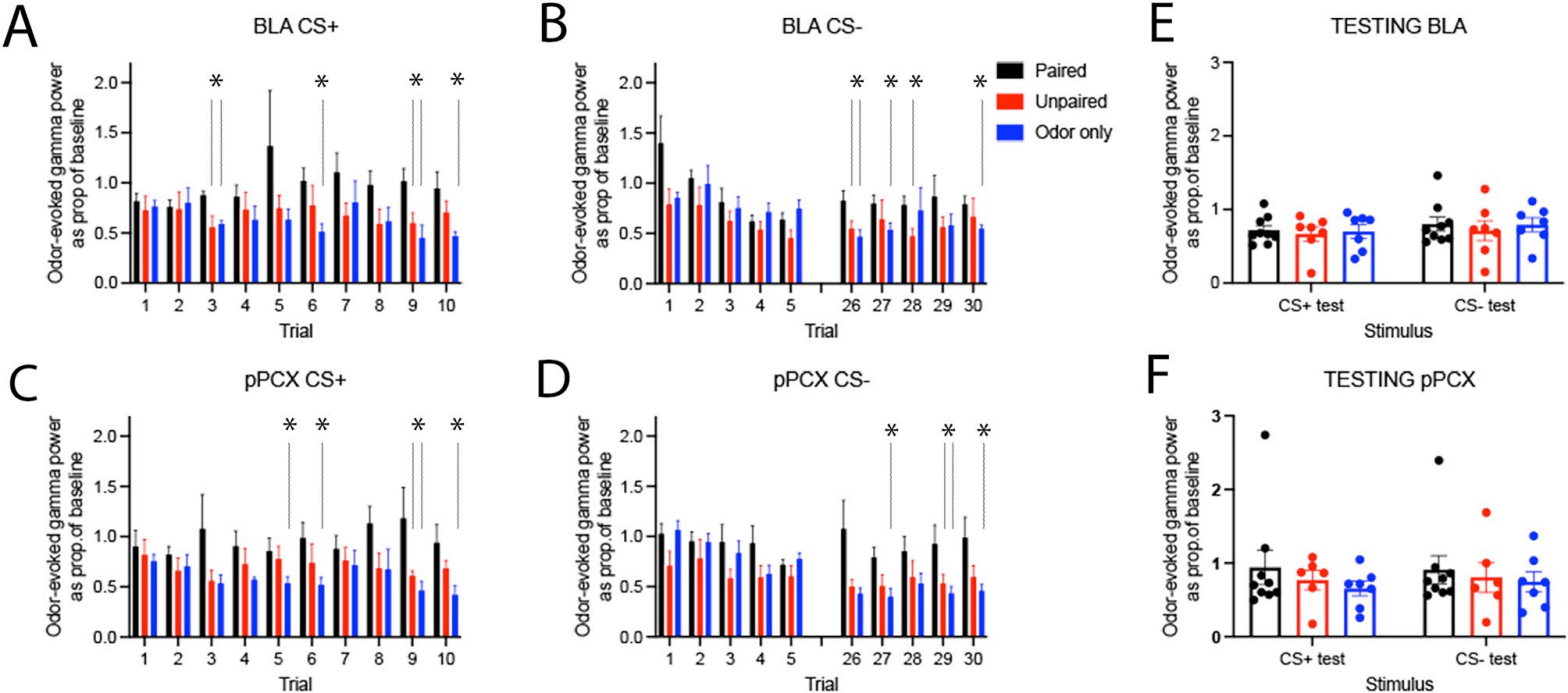
Normalized odor-evoked high gamma response for each of the first 5 trials and last 5 trials of the CS+ and CS− in both regions. The CS + was delivered 10 times and the CS− was deliver 30 times. High gamma was significantly greater in Paired animals than controls in both the BLA (**A**, **B**) and the pPCX (**C**, **D**), with differences emerging within the first 5 trials for the CS+ but not until later trials for the CS− (asterisks signify significantly larger responses in the Paired animals compared to specific controls as identified with vertical lines, *p* < 0.05). No differences were observed during testing in either the BLA (**E**) or pPCX (**F**).

As shown in Fig. 3, odor-evoked oscillatory activity was modified in both the BLA and pPCX over the course of training relative to controls. For the CS+ , BLA high gamma band (61–90 Hz) response was significantly enhanced by the end of training in Paired animals compared to Controls (repeated ANOVA, group X trial, main effect of group F(2,21) = 3.87, *p* = 0.037, post-hoc Fisher tests revealed significantly larger gamma responses in Paired animals compared to controls on trials as indicated in Fig. 3). A similar trend was observed in the pPCX response to the CS+ (repeated ANOVA, group X trial, main effect of group F(2,20) = 3.22, *p* = 0.06, post-hoc Fisher tests revealed significantly larger gamma responses in Paired animals compared to controls as indicated in Fig. 3).

Similar elevated gamma oscillations were seen in the BLA and PCX in response to CS−. In the BLA there was a significant effect of Trial (F(4,72 = 4.03, *p* = 0.006) and a main effect of Group (F(2,21) = 3.94, p = 0.035). Post-hoc tests revealed significantly larger gamma responses in Paired animals compared to controls as indicated in Fig. 3. Similarly, in the pPCX there was a significant effect of Group (F(2,20 = 4.71, *p* = 0.021) and Trial interaction (F(4,65) = 2.84, *p* = 0.036). Post-hoc tests revealed significant differences between groups as indicated in Fig. 3. No significant changes in gamma activity were observed when the last 5 s of the CS odors (immediately preceding the shock [CS+] or no shock [CS−] events) was examined (data not shown).

Across both regions and stimuli, the effects were primarily driven by reductions in gamma band odor-evoked responses over the course of training in Control animals and relatively maintained responses in Paired animals (Fig. 3). During testing 24 h after training, there were no significant differences between Paired and Control odor-evoked gamma to either the CS+ or CS− in either the BLA or pPCX (Fig. 3E and F).

**Figure 4:**
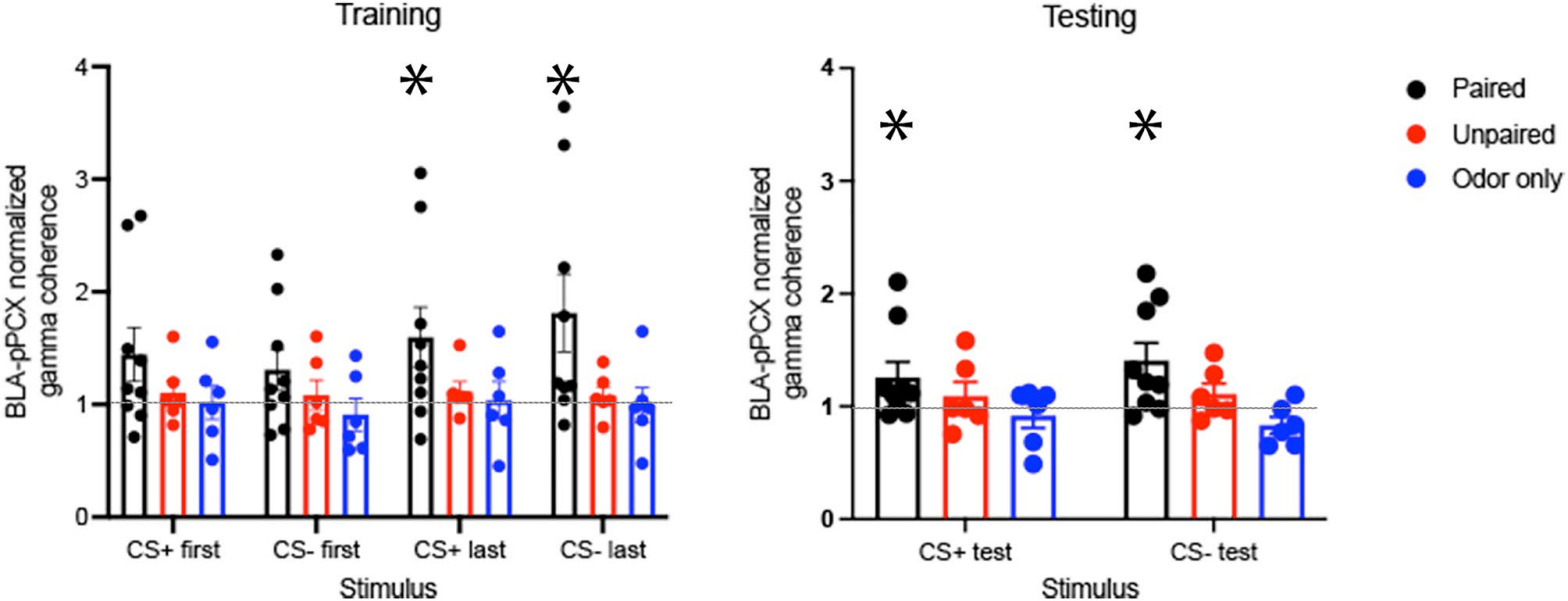
Odor-evoked BLA-pPCX LFP coherence in the high gamma band normalized to spontaneous coherence in the homecage. During training, coherence was significantly greater in Paired animals than either control group (ANOVA group X stimulus interaction, *p* < 0.05) with the largest differences expressed over the last 5 trials of the CS+ and CS−. The odor-evoked BLA-pPCX coherence was enhanced during training and test compared to that observed in the homecage only in Paired animals (asterisks signifies significant difference from 1.0, *p* < 0.05) and not in either control groups.

While odor evoked activity was modified in both BLA and pPCX, was functional connectivity between these regions changed by odor threat conditioning? BLA-pPCX gamma coherence during the first 5 s of CS+ and CS− odors was expressed as a proportion of gamma coherence recorded in the home cage (Fig. 4). There was no significant difference between coherence in the home cage between Paired, Unpaired and Odor-only rats (ANOVA, *p* = 0.36, NS). Normalized BLA-pPCX gamma coherence increased over the course of paired conditioning compared to controls (3 × 4, group X stimulus ANOVA, main effect of stimulus, F(6,54) = 3.05, *p* = 0.012, group X stimulus interaction, F(1,25) = 3.99, *p* = 0.044). Planned post-hoc tests to determine whether coherence had significantly changed from that observed in the home cage showed that BLA-pPCX coherence during the last five presentations of the CS+ and CS− were significantly enhanced only in the Paired animals. Paired animals showed normalized odor-evoked coherence significantly greater than 1 during the last 5 trials to both the CS+ and CS− (t-tests, *p* < 0.05), while Unpaired and Odor-only controls did not, suggesting an enhancement in connectivity over the course of training. Twenty four hours post conditioning, when tested in a novel context, BLA-pPCX gamma coherence was significantly enhanced to both the CS+ and CS− in Paired animals compared to Controls (3 X 2 group X stimulus interaction, ANOVA, main effect of group, F(2,18) = 3.72, *p* = 0.044). Paired animals showed normalized odor-evoked coherence significantly greater than 1 during testing to both the CS+ and CS− (t-tests, *p* < 0.05), suggesting maintained enhancement in BLA-pPCX connectivity during testing, while neither control group showed enhanced coherence.

Together, these results demonstrate a learning-induced change in odor-evoked activity in both the BLA and pPCX, though in this paradigm these regional changes were not detected at testing. In contrast, threat conditioning also enhanced functional connectivity between the BLA and pPCX, which was maintained for at least 24 h. While there is extensive evidence that the BLA is required for acquisition, and at least initial periods of expression of learned responses to threat^55,56^, the focus here is on whether both the BLA and pPCX play critical roles in odor threat conditioning, and the specific role of BLA-pPCX interaction in this learned behavior. Thus, as an important next step we simply asked whether the pPCX was necessary for normal odor threat conditioning.

**Figure 5:**
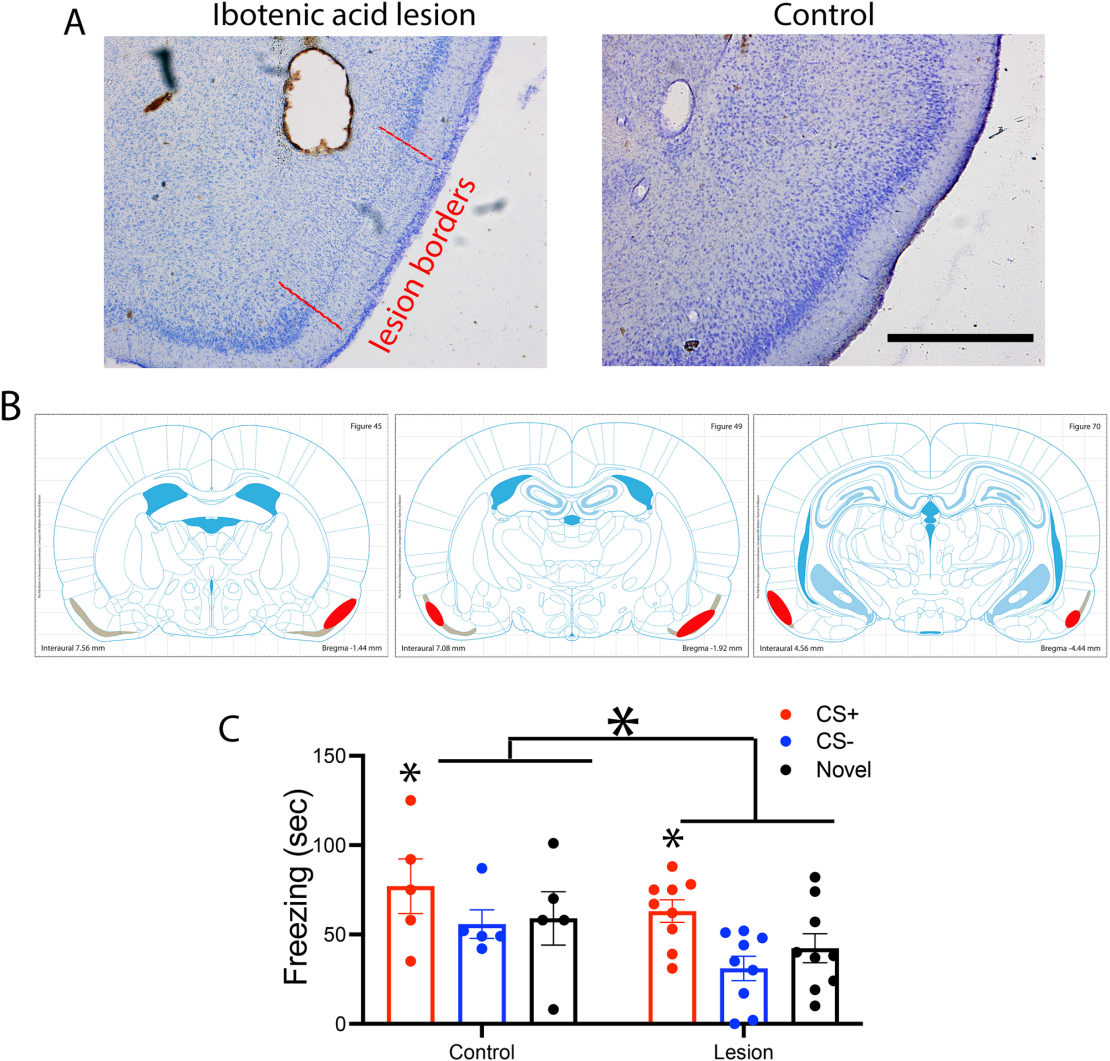
(**A**) Representative ibotenic acid lesion of the pPCX from a single animal and representative control. Ibotenic acid lesion induced cell loss in regions of pPCX layer II are highlighted with red lines. Scale bar = 1 mm. (**B**) Reconstruction of the extent of lesion in one animal. Gray zone is Layer II of the pPCX. Red areas highlight regions of cell loss. Schematic brain images were modified from Paxinos and Watson^77^. (**C**) Animals with partial cell loss in pPCX showed a significant reduction in odor-evoked freezing after odor threat conditioning. Small asterisks signify significantly more freezing to the CS+ than other odors in both the Control and Lesion groups (*p* < 0.05). Large asterisk signifies significantly less freezing in the Lesioned group compared to Control (ANOVA main effect of group, *p* < 0.05).

The pPCX is necessary for olfactory threat conditioning. Ibotenic acid infusions into the pPCX induced robust cell loss in layer II and III as shown in Fig. 5A and B. Control animals infused with saline showed no signs of cell loss. These excitotoxic pPCX lesions prior to odor threat conditioning (10 CS+ [vanilla] pairings with footshock and 30 CS− [peppermint] presentations) significantly reduced odor-evoked freezing in a novel context (Fig. 5C). Odor-evoked freezing was reduced to both the CS+ and CS−, as well as to a novel control odor (isoamyl acetate) presented only during testing (Fig. 5C). A 2 X 3 ANOVA (group X stimulus) showed a significant main effect of both group and stimulus (group, F(1,42) = 5.53, *p* = 0.024; stimulus, F(2,42) = 4.12, *p* = 0.025). Post-hoc tests revealed freezing was significantly greater to the CS+ than the other odors in both the control (n = 5) and lesioned (n = 9) animals, though overall odor-evoked freezing was less in lesioned rats. pPCX lesioned animals were not anosmic as determined with a buried food task. All control and lesioned animals tested found the food within the 300 s trial (latency to find and retrieve food, controls = 163 ± 35 s, pPCX lesioned = 165.5 ± 15.5 s). Furthermore there was no effect on activity levels in an open field test (total distance traveled, controls = 20.5 ± 2.4 m; pPCX lesion = 21.2 ± 2.7 m, t-test, t(12) = 0.18, *p* = 0.85). These data further demonstrate a critical role for the pPCX in odor threat conditioning^57^.

Thus, odor threat conditioning enhances functional connectivity between the BLA and pPCX, and an intact pPCX is important for full acquisition/expression of learned odor threat. We next examined whether pPCX involvement in odor threat conditioning was related specifically to its input from the BLA.

**Figure 6:**
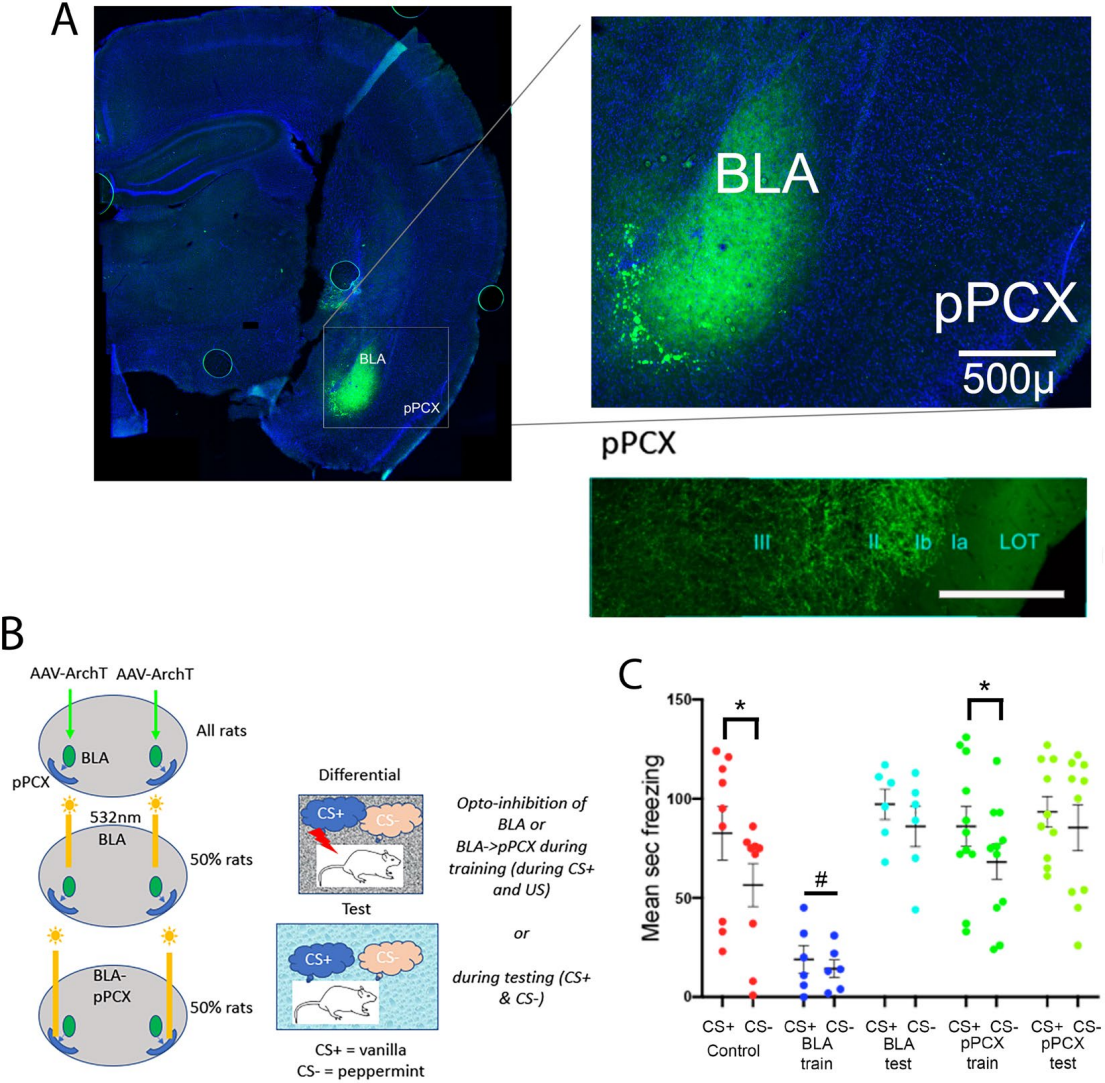
(**A**) Example of AAV5-CaMKIIa-eArchT3.0-eYFP infusion into the BLA. Animal was perfused 2 months after the experiment was completed, thus some viral over-expression is observed. Optical fiber track is not in this section. Lower right panel shows YFP labels BLA fibers within pPCX. Scale in inset = 100µ. (**B**) Experimental design. AAV5-CaMKIIa-eArchT3.0-eYFP, or control virus, was infused bilaterally in the BLA. Optical fibers were implanted bilaterally targeting either the BLA or BLA fibers within the pPCX to allow suppression of activity within the BLA or selectively BLA projections to the pPCX during odor threat conditioning or testing. (**C**) Odor-evoked freezing to the CS+ and CS− in each group. Viral/light Controls froze significantly more to the CS+ than the CS−, as did animals that had BLA projections to the pPCX suppressed during training (asterisks signify significant difference between CS+ and CS−, *p* < 0.05). Suppression of the BLA during training blocked threat conditioning (# signifies significant difference from all other groups, *p* < 0.05). Suppression of the BLA, or selectively of the BLA projection to the pPCX during testing, however, did not impair expression of CS+ evoked freezing, but did enhance generalization to the CS−. There were no differences in odor-evoked freezing between the CS+ and CS− in these groups.

BLA input to pPCX is necessary for odor-specific learned threat response. Animals (n = 67) received bilateral infections of AAV5-CaMKIIa-eArchT3.0-eYFP to the BLA (Fig. 6A) and bilateral optical fibers implanted targeting either the BLA or the pPCX (Fig. 6B). Optogenetic controls included control virus infections (AAV5-CaMKIIa-eYFP) or AAV5-CaMKIIa-eArchT3.0-eYFP infections with no light applied (no difference in behavioral outcomes was detected between these control groups). Following histological confirmation of infections limited to the bilateral BLA, data from 42 animals were included in the analysis (Control n = 9; BLA suppression during acquisition n = 6; BLA suppression during testing n = 6; BLA fiber suppression within pPCX during acquisition n = 11; BLA fiber suppression within pPCX during testing n = 10). Following at least 4 weeks of recovery, the different optical fiber placements allowed suppression of either neurons within the BLA and all of their projections, or selective suppression of BLA projections to the pPCX during odor threat conditioning or during odor threat expression. Animals were conditioned as in the protocols above in a differential odor threat conditioning paradigm with a CS+ odor signaling shock and a CS− odor signaling safety. In some animals, activity in CAMKII expressing BLA neurons was suppressed with 532 nm light (> 8mW) during CS+/shock exposure during training while other animals were trained without optogenetic suppression and instead had suppression imposed during testing stimulus exposure.

As shown in Fig. 6C, controls animals learned an odor specific freezing response, showing significantly greater freezing to the CS+ than to the CS−. In contrast, optogenetic suppression of BLA activity during the CS+/shock presentations during training blocked acquisition of a learned odor threat response. Selectively suppressing BLA projections to the pPCX during training, however, had no significant effect on odor specific threat learning, with animals showing significant freezing to the CS+ and significantly less freezing to the CS−. This suggests that BLA input to the pPCX is not necessary for acquisition of normal odor specific threat responses.

However, suppression of either the BLA or selectively of the BLA projection to the pPCX during testing disrupted expression of normal odor-specific threat responses. Animals in both groups showed normal levels of freezing to the CS+ , but showed non-specific, generalized freezing to the CS− (2-way ANOVA, optical manipulation X stimulus, main effect of stimulus, F(1,37) = 21.17, *p* < 0.0001; main effect of optical manipulation, F(4,37) = 7.48, *p* = 0.0002; post-hoc tests revealed CS+ vs CS− differences in Control, *p* = 0.0007 and pPCX training, *p* = 0.014, but not between other stimuli in other groups).

## Discussion

The present results demonstrate that the BLA and pPCX function in concert to allow odor-specific learned threat responses^58^. Specifically, both optogenetic suppression of BLA activity and pPCX lesions impaired learned threat acquisition, suggesting that both regions are critical for normal odor threat conditioning and/or expression. Dual BLA and pPCX LFP recordings during differential odor threat conditioning was used to better understand this cross talk. Specifically, there was enhanced odor-evoked high gamma band coherence between the BLA and pPCX both during acquisition and during testing 24 h later, suggesting increased connectivity between these regions as acquisition progressed, which continued after conditioning through testing the next day. This enhanced coherence was expressed to both the CS+ and the CS−, suggesting information about both the learned threat odor and the learned safety odor was expressed in this circuit^59^. Although selective suppression of the BLA input to the pPCX during training did not affect odor-specific learned fear, suppressing that connectivity during testing, either by optogenetic suppression of the BLA or by selective suppression of BLA projections to the pPCX induced generalized odor threat responses, rather than odor specific threat. These results suggest that a critical role of BLA input to the pPCX is either to enhance olfactory acuity during expression allowing differential behavioral responses to odors, or to assign threat/safety valence to odor representations within the pPCX, again to allow differential odor responses. Additional research will be required to distinguish between these two mechanisms; however, prior work has demonstrated that BLA input modifies single-unit ensemble odor coding within pPCX^47^, suggesting some BLA modulation of pPCX odor processing.

Odor threat conditioning enhanced high gamma band LFP oscillations in both the BLA and pPCX during acquisition and enhanced high gamma band BLA-pPCX coherence during acquisition and expression. Previous work has demonstrated that odor threat conditioning induces changes in odor-evoked activity in both the BLA^25,41,60^ and PCX^25,26,29,43^, among other regions^12,48,61^,which can be long-lasting. It is unclear why the paradigm used here did not induce changes in LFP odor-evoked responses that were expressed 24 h after conditioning when tested in a novel context. However, BLA-pPCX coherence was maintained throughout both acquisition and expression, suggesting a lasting change in this circuit selectively induced by threat conditioning. In addition, both learned and innate aversive odors have been reported to induce changes in a variety of LFP oscillation frequencies in including theta, beta, and gamma band in the olfactory system, amygdala or other related structures^29,43,62,63^. The LFP results reported here were primarily within the gamma band which replicates previous work showing enhanced gamma oscillations in the PCX during this odor fear conditioning protocol^43^. BLA gamma oscillations have been shown to be robust and sensitive to sensory input^64^ and in the olfactory system can be indicative of a period of high synchrony in neural firing which is an effective way to drive downstream targets^63^. The present results also strengthen previous data demonstrating that gamma oscillation amplitude is sensitive to odor learning and experience^43,63,65^.

Activity within the BLA encodes both threat and safety^33,66^, and distributed BLA output projections can drive either threat- or reward-appropriate behavioral responses via the central nucleus of the amygdala or nucleus accumbens, respectively^59^. In the auditory system where it has been most thoroughly investigated, the stimulus specificity of these learned behaviors has been shown to be dependent on the specificity of sensory inputs, either via the thalamus or sensory neocortex^67–69^. Auditory threat conditioning modifies tone evoked responses and receptive fields of auditory cortical neurons^67,70,71^ and auditory system lesions^72^, or optogenetic suppression of auditory cortical input to the lateral amygdala^67^ impaired acquisition and expression of stimulus-selective fear. However, there are also projections from the amygdala to the auditory cortex^73^, and those projections are important in shaping long latency learned responses of auditory cortical neurons to threatening tones^70^.

In contrast to the auditory system, however, the reciprocal connectivity between the amygdala and the olfactory pathway is much more robust, with, for example, direct connections between the BLA and the full anterior–posterior extent of the piriform and lateral entorhinal cortex^45,74,75^. BLA fibers target both pyramidal cells and interneurons within PCX^46^. The pPCX, considered a form of association cortex^76^, receives the strongest BLA input^45^, and optogenetic activation of BLA fibers within the pPCX modulates odor coding^47^. The present results suggest that this BLA input is required for expression of odor-specific behavioral threat responses, presumably via modulation of pPCX ensemble responses to both the threat and safety odors. BLA neurons projecting to the lateral entorhinal cortex show enhanced responses to threatening stimuli after conditioning^75^, thus a similar mechanism could occur in the pPCX to modify responses to the CS+ and CS−^47^. Differential odor threat conditioning has been shown to modify PCX single-unit responses to the CS+ and CS− in freely moving rats in a manner that should enhance discriminability of the two odors^26^. Whether this PCX odor coding change is due to BLA input, and whether it underlies the observed loss of behavioral odor specificity after BLA input suppression is currently being examined. Nonetheless, the results suggest that in contrast to auditory threat conditioning, stimulus specificity in the behavioral expression of odor threat involves a direct BLA modulation of the sensory cortex.

In summary, the present results suggest that BLA-pPCX connectivity is enhanced by differential odor threat conditioning, and that suppression of BLA input to the pPCX impairs expression of behavioral response odor specificity, resulting in threat generalization. This BLA-sensory cortex inter-relationship appears to differ from the auditory system, reflecting the unique anatomical relationship between the amygdala and the olfactory cortex.

## References

[1] Davis RG (1981). The role of nonolfactory context cues in odor identification. Percept. Psychophys..

[2] Spence C (2020). Olfactory-colour crossmodal correspondences in art, science, and design. Cogn. Res. Princ. Implic..

[3] Stevenson RJ, Oaten M (2008). The effect of appropriate and inappropriate stimulus color on odor discrimination. Percept. Psychophys..

[4] Gottfried JA, Dolan RJ (2003). The nose smells what the eye sees: Crossmodal visual facilitation of human olfactory perception. Neuron.

[5] Mandairon N, Kermen F, Charpentier C, Sacquet J, Linster C, Didier A (2014). Context-driven activation of odor representations in the absence of olfactory stimuli in the olfactory bulb and piriform cortex. Front. Behav. Neurosci..

[6] Olofsson, J. K., Zhou, G., East, B. S., Zelano, C. & Wilson, D. A. Odor identification in rats: Behavioral and electrophysiological evidence of learned olfactory-auditory associations. *eNeuro* **6**, (2019). 10.1523/ENEURO.0102-19.2019 PMC6709214 31362955

[7] Porada DK, Regenbogen C, Seubert J, Freiherr J, Lundstrom JN (2019). Multisensory enhancement of odor object processing in primary olfactory cortex. Neuroscience.

[8] Zhou G, Lane G, Noto T, Arabkheradmand G, Gottfried JA, Schuele SU, Rosenow JM, Olofsson JK, Wilson DA, Zelano C (2019). Human olfactory-auditory integration requires phase synchrony between sensory cortices. Nat. Commun..

[9] Kontaris I, East BS, Wilson DA (2020). Behavioral and neurobiological convergence of odor, mood and emotion: A review. Front. Behav. Neurosci..

[10] Pager J, Giachetti I, Holley A, Le Magnen J (1972). A selective control of olfactory bulb electrical activity in relation to food deprivation and satiety in rats. Physiol. Behav..

[11] Palouzier-Paulignan B, Lacroix MC, Aime P, Baly C, Caillol M, Congar P, Julliard AK, Tucker K, Fadool DA (2012). Olfaction under metabolic influences. Chem. Senses.

[12] Perry RE, Al Ain S, Raineki C, Sullivan RM, Wilson DA (2016). Development of odor hedonics: experience-dependent ontogeny of circuits supporting maternal and predator odor responses in rats. J. Neurosci..

[13] Rudell JB, Rechs AJ, Kelman TJ, Ross-Inta CM, Hao S, Gietzen DW (2011). The anterior piriform cortex is sufficient for detecting depletion of an indispensable amino acid, showing independent cortical sensory function. J. Neurosci..

[14] Bensafi M, Sobel N, Khan RM (2007). Hedonic-specific activity in piriform cortex during odor imagery mimics that during odor perception. J. Neurophysiol..

[15] Chapuis J, Wilson DA (2011). Bidirectional plasticity of cortical pattern recognition and behavioral sensory acuity. Nat. Neurosci..

[16] Manabe H, Kusumoto-Yoshida I, Ota M, Mori K (2011). Olfactory cortex generates synchronized top-down inputs to the olfactory bulb during slow-wave sleep. J Neurosci..

[17] Wang D, Liu P, Mao X, Zhou Z, Cao T, Xu J, Sun C, Li A (2019). Task-demand-dependent neural representation of odor information in the olfactory bulb and posterior piriform cortex. J. Neurosci..

[18] Wilson DA (2010). Single-unit activity in piriform cortex during slow-wave state is shaped by recent odor experience. J. Neurosci..

[19] Lundstrom JN, Olsson MJ (2005). Subthreshold amounts of social odorant affect mood, but not behavior, in heterosexual women when tested by a male, but not a female, experimenter. Biol. Psychol..

[20] Sullivan RM, Wilson DA (1993). Role of the amygdala complex in early olfactory associative learning. Behav. Neurosci..

[21] Krusemark EA, Novak LR, Gitelman DR, Li W (2013). When the sense of smell meets emotion: Anxiety-state-dependent olfactory processing and neural circuitry adaptation. J. Neurosci..

[22] Herz RS (2009). Aromatherapy facts and fictions: A scientific analysis of olfactory effects on mood, physiology and behavior. Int. J. Neurosci..

[23] Calu DJ, Roesch MR, Stalnaker TA, Schoenbaum G (2007). Associative encoding in posterior piriform cortex during odor discrimination and reversal learning. Cereb Cortex.

[24] Chandra, N., Awasthi, R., Ozdogan, T., Johenning, F. W., Imbrosci, B., Morris, G., Schmitz, D. & Barkai, E. A cellular mechanism underlying enhanced capability for complex olfactory discrimination learning. *eNeuro* **6**, (2019). 10.1523/ENEURO.0198-18.2019 PMC6378325 30783614

[25] Chapuis J, Garcia S, Messaoudi B, Thevenet M, Ferreira G, Gervais R, Ravel N (2009). The way an odor is experienced during aversive conditioning determines the extent of the network recruited during retrieval: A multisite electrophysiological study in rats. J. Neurosci..

[26] Chen CF, Barnes DC, Wilson DA (2011). Generalized vs. stimulus-specific learned fear differentially modifies stimulus encoding in primary sensory cortex of awake rats. J. Neurophysiol..

[27] Cohen Y, Wilson DA, Barkai E (2015). Differential modifications of synaptic weights during odor rule learning: Dynamics of interaction between the piriform cortex with lower and higher brain areas. Cereb Cortex.

[28] Dupin, M., Garcia, S., Boulanger-Bertolus, J., Buonviso, N. & Mouly, A. M. New insights from 22-kHz ultrasonic vocalizations to characterize fear responses: relationship with respiration and brain oscillatory dynamics. *eNeuro* **6**, (2019). 10.1523/ENEURO.0065-19.2019 PMC6506822 31064837

[29] Dupin M, Garcia S, Messaoudi B, Doyere V, Mouly AM (2020). Respiration and brain neural dynamics associated with interval timing during odor fear learning in rats. Sci. Rep..

[30] Porter DB, Qu LP, Kahnt T, Gottfried JA (2021). Aversive outcomes impact human olfactory discrimination learning and generalization. Behav. Neurosci..

[31] Roesch MR, Stalnaker TA, Schoenbaum G (2007). Associative encoding in anterior piriform cortex versus orbitofrontal cortex during odor discrimination and reversal learning. Cereb Cortex.

[32] Kyriazi P, Headley DB, Pare D (2018). Multi-dimensional coding by basolateral amygdala neurons. Neuron.

[33] Likhtik E, Stujenske JM, Topiwala MA, Harris AZ, Gordon JA (2014). Prefrontal entrainment of amygdala activity signals safety in learned fear and innate anxiety. Nat. Neurosci..

[34] Morikawa S, Katori K, Takeuchi H, Ikegaya Y (2021). Brain-wide mapping of presynaptic inputs to basolateral amygdala neurons. J. Comp. Neurol..

[35] Otto T, Giardino ND (2001). Pavlovian conditioning of emotional responses to olfactory and contextual stimuli: A potential model for the development and expression of chemical intolerance. Ann. N. Y. Acad. Sci..

[36] Sangha S, Diehl MM, Bergstrom HC, Drew MR (2020). Know safety, no fear. Neurosci. Biobehav. Rev..

[37] Warlow SM, Naffziger EE, Berridge KC (2020). The central amygdala recruits mesocorticolimbic circuitry for pursuit of reward or pain. Nat. Commun..

[38] Zhang X, Kim J, Tonegawa S (2020). Amygdala reward neurons form and store fear extinction memory. Neuron.

[39] Cousens G, Otto T (1998). Both pre- and posttraining excitotoxic lesions of the basolateral amygdala abolish the expression of olfactory and contextual fear conditioning. Behav. Neurosci..

[40] Cain DP, Bindra D (1972). Responses of amygdala single units to odors in the rat. Exp. Neurol..

[41] Rosenkranz JA, Grace AA (2002). Dopamine-mediated modulation of odour-evoked amygdala potentials during pavlovian conditioning. Nature.

[42] Sullivan RM, Landers M, Yeaman B, Wilson DA (2000). Good memories of bad events in infancy. Nature.

[43] Barnes DC, Chapuis J, Chaudhury D, Wilson DA (2011). Odor fear conditioning modifies piriform cortex local field potentials both during conditioning and during post-conditioning sleep. PLoS ONE.

[44] Cleland, T. A. & Linster, C. in *Handbook of olfaction and gustation* (ed R. L. Doty) 165–180 (Marcel Dekker, 2003).

[45] Majak K, Ronkko S, Kemppainen S, Pitkanen A (2004). Projections from the amygdaloid complex to the piriform cortex: A PHA-L study in the rat. J. Comp. Neurol..

[46] Luna VM, Morozov A (2012). Input-specific excitation of olfactory cortex microcircuits. Front. Neural Circ..

[47] Sadrian B, Wilson DA (2015). Optogenetic stimulation of lateral amygdala input to posterior piriform cortex modulates single-unit and ensemble odor processing. Front. Neural Circ..

[48] Kass MD, Rosenthal MC, Pottackal J, McGann JP (2013). Fear learning enhances neural responses to threat-predictive sensory stimuli. Science.

[49] Parma V, Ferraro S, Miller SS, Ahs F, Lundstrom JN (2015). Enhancement of odor sensitivity following repeated odor and visual fear conditioning. Chem. Senses.

[50] Li W, Howard JD, Parrish TB, Gottfried JA (2008). Aversive learning enhances perceptual and cortical discrimination of indiscriminable odor cues. Science.

[51] Kilkenny C, Browne WJ, Cuthill IC, Emerson M, Altman DG (2010). Improving bioscience research reporting: The ARRIVE guidelines for reporting animal research. PLoS Biol..

[52] Alberts JR, Galef BG (1971). Acute anosmia in the rat: A behavioral test of a peripherally-induced olfactory deficit. Physiol. Behav..

[53] Courtiol E, Neiman M, Fleming G, Teixeira CM, Wilson DA (2019). A specific olfactory cortico-thalamic pathway contributing to sampling performance during odor reversal learning. Brain Struct. Funct..

[54] Mahn M, Prigge M, Ron S, Levy R, Yizhar O (2016). Biophysical constraints of optogenetic inhibition at presynaptic terminals. Nat. Neurosci..

[55] Cahill L, Weinberger NM, Roozendaal B, McGaugh JL (1999). Is the amygdala a locus of "conditioned fear"? Some questions and caveats. Neuron.

[56] Fanselow MS, LeDoux JE (1999). Why we think plasticity underlying Pavlovian fear conditioning occurs in the basolateral amygdala. Neuron.

[57] Sacco T, Sacchetti B (2010). Role of secondary sensory cortices in emotional memory storage and retrieval in rats. Science.

[58] Hegoburu C, Parrot S, Ferreira G, Mouly AM (2014). Differential involvement of amygdala and cortical NMDA receptors activation upon encoding in odor fear memory. Learn. Mem..

[59] O'Neill PK, Gore F, Salzman CD (2018). Basolateral amygdala circuitry in positive and negative valence. Curr. Opin. Neurobiol..

[60] Raineki C, Cortes MR, Belnoue L, Sullivan RM (2012). Effects of early-life abuse differ across development: infant social behavior deficits are followed by adolescent depressive-like behaviors mediated by the amygdala. J. Neurosci..

[61] Ross JM, Fletcher ML (2018). Learning-dependent and -independent enhancement of mitral/tufted cell glomerular odor responses following olfactory fear conditioning in awake mice. J. Neurosci..

[62] Heale VR, Vanderwolf CH (1994). Dentate gyrus and olfactory bulb responses to olfactory and noxious stimulation in urethane anaesthetized rats. Brain Res..

[63] Kay LM (2014). Circuit oscillations in odor perception and memory. Prog. Brain Res..

[64] Headley, D. B., Kyriazi, P., Feng, F., Nair, S. & Pare, D. Gamma oscillations in the basolateral amygdala: Localization, microcircuitry, and behavioral correlates. *J. Neurosci.*, (2021). 10.1523/JNEUROSCI.3159-20.2021 PMC8276735 34088799

[65] Martin C, Ravel N (2014). Beta and gamma oscillatory activities associated with olfactory memory tasks: Different rhythms for different functional networks?. Front. Behav. Neurosci..

[66] Tzovara A, Meyer SS, Bonaiuto JJ, Abivardi A, Dolan RJ, Barnes GR, Bach DR (2019). High-precision magnetoencephalography for reconstructing amygdalar and hippocampal oscillations during prediction of safety and threat. Hum. Brain Mapp..

[67] Dalmay T, Abs E, Poorthuis RB, Hartung J, Pu DL, Onasch S, Lozano YR, Signoret-Genest J, Tovote P, Gjorgjieva J, Letzkus JJ (2019). A critical role for neocortical processing of threat memory. Neuron.

[68] Romanski LM, LeDoux JE (1992). Equipotentiality of thalamo-amygdala and thalamo-cortico-amygdala circuits in auditory fear conditioning. J. Neurosci..

[69] Wigestrand MB, Schiff HC, Fyhn M, LeDoux JE, Sears RM (2017). Primary auditory cortex regulates threat memory specificity. Learn. Mem..

[70] Armony JL, Quirk GJ, LeDoux JE (1998). Differential effects of amygdala lesions on early and late plastic components of auditory cortex spike trains during fear conditioning. J. Neurosci..

[71] Weinberger NM (1998). Physiological memory in primary auditory cortex: Characteristics and mechanisms. Neurobiol. Learn. Mem..

[72] Jarrell TW, Gentile CG, Romanski LM, McCabe PM, Schneiderman N (1987). Involvement of cortical and thalamic auditory regions in retention of differential bradycardiac conditioning to acoustic conditioned stimuli in rabbits. Brain Res..

[73] Bertero A, Feyen PLC, Zurita H, Apicella AJ (2019). A non-canonical cortico-amygdala inhibitory loop. J. Neurosci..

[74] Ferry B, Wirth S, Di Scala G (1999). Functional interaction between entorhinal cortex and basolateral amygdala during trace conditioning of odor aversion in the rat. Behav. Neurosci..

[75] Majak K, Pitkanen A (2003). Activation of the amygdalo-entorhinal pathway in fear-conditioning in rat. Eur. J. Neurosci..

[76] Haberly LB (2001). Parallel-distributed processing in olfactory cortex: New insights from morphological and physiological analysis of neuronal circuitry. Chem. Senses.

[77] Paxinos G, Watson C (2009). The Rat Brain in Stereotaxic Coordinates.

